# Proprotein Convertase Subtilisin/Kexin Type 9 (PCSK9) in the Brain and Relevance for Neuropsychiatric Disorders

**DOI:** 10.3389/fnins.2020.00609

**Published:** 2020-06-12

**Authors:** Emma M. O’Connell, Falk W. Lohoff

**Affiliations:** Section on Clinical Genomics and Experimental Therapeutics, National Institute on Alcohol Abuse and Alcoholism, National Institutes of Health, Bethesda, MD, United States

**Keywords:** PCSK9, LDLR, brain, Alzheimer’s disease, alcohol use disorder, stroke, neurocognition, neuroinflammation

## Abstract

Proprotein convertase subtilisin/kexin type 9 (PCSK9) has long been studied in the liver due to its regulation of plasma low-density lipoprotein cholesterol (LDL-C) and its causal role in familial hypercholesterolemia. Although PCSK9 was first discovered in cerebellar neurons undergoing apoptosis, its function in the central nervous system (CNS) is less clear. PCSK9 has been shown to be involved in neuronal differentiation, LDL receptor family metabolism, apoptosis, and inflammation in the brain, but *in vitro* and *in vivo* studies offer contradictory findings. PCSK9 expression in the adult brain is low but is highly upregulated during disease states. Cerebral spinal fluid (CSF) PCSK9 concentrations are correlated with neural tube defects and neurodegenerative diseases in human patients. Epigenetic studies reveal that chronic alcohol use may modulate methylation of the *PCSK9* gene and genetic studies show that patients with gain-of-function *PCSK9* variants have higher LDL-C and an increased risk of ischemic stroke. Early safety studies of the PCSK9 inhibitors evolocumab and alirocumab, used to treat hypercholesterolemia, hinted that PCSK9 inhibition may negatively impact cognition but more recent, longer-term clinical trials found no adverse neurocognitive events. The purpose of this review is to elucidate the role of PCSK9 in the brain, particularly its role in disease pathogenesis.

## Introduction

Proprotein convertase subtilisin/kexin type 9 (PCSK9) was first identified in 2003 in primary cerebellar neurons as a mRNA upregulated during apoptosis (Chiang et al., 2001; Seidah et al., 2003). Originally called neural apoptosis-regulated convertase-1 (NARC-1), PCSK9 is the ninth member of the mammalian family of serine proteinases, a group of protein convertases (PCs) that cleave inactive secretory precursors into bioactive proteins and peptides. The discovery of PCSK9 was driven by the existence of non-basic amino acid processing sites not recognized by known PCs (Seidah et al., 2003). The human *PCSK9* gene is located on chromosome 1p32.3 and is translated into a ∼82-kDa zymogen in the endoplasmic reticulum (ER) (Abifadel et al., 2003; Piper et al., 2007). The PCSK9 pro-form is autocatalytically cleaved at its internal VFAQ_152_ sequence into mature PCSK9 in the ER. It is secreted as a heterodimer protein with its ∼17 kDa prodomain still bound to its catalytic domain to inhibit its catalytic activity (Seidah et al., 2003; Benjannet et al., 2004; Piper et al., 2007; Seidah and Prat, 2012). PCSK9 is mainly secreted by hepatocytes into the blood stream and exists in the plasma in an active and inactive form. The active form consists of a full-length heterodimer (∼62 kDa) that is predominantly associated with the low-density lipoprotein (LDL) particle, which protects PCSK9 from being cleaved by furin into its inactive form. The inactive heterodimer (∼55 kDa), representing 15–40% of total circulating PCSK9, circulates freely and has at least a twofold lower affinity to LDLR and a limited ability to degrade it (Tavori et al., 2013; Shapiro et al., 2018; Macchi et al., 2019). PCSK9 mainly interacts with LDL and may marginally interact with high-density lipoprotein (HDL), although findings are controversial (Kosenko et al., 2013; Ferri et al., 2016a; Burnap et al., 2020).

The most prominent role of PCSK9 is its interaction with the low-density lipoprotein receptor (LDLR) in the liver, which was discovered in 2003 in a French family with autosomal dominant hypercholesterolemia who had two gain-of-function mutations in the *PCSK9* gene (Abifadel et al., 2003). When an LDL particle with PCSK9 binds to an LDLR, the catalytic domain of PCSK9 interacts with the epidermal growth factor-like repeat A (EGF-A) domain of the LDLR. The low pH of the endosome enhances PCSK9/LDLR affinity when the complex is endocytosed, and PCSK9 prevents the open extended conformation of LDLR associated with receptor recycling. Instead, the PCSK9/LDLR complex is shuttled to the lysosome for degradation, resulting in fewer surface LDLRs and higher plasma cholesterol levels (Seidah et al., 2003; Benjannet et al., 2004; Poirier et al., 2006; Lo Surdo et al., 2011). Regulation of plasma PCSK9, LDLR, and LDL-C levels is tightly linked because PCSK9 is cleared from the plasma mainly by binding to LDLR but at the same time induces LDLR degradation due to its interaction (Tavori et al., 2013).

PCSK9 interacts with several receptors in the LDL receptor family. While PCSK9 mainly interacts with LDLR in the liver (Lagace et al., 2006; Grefhorst et al., 2008), it also binds to the LDL receptor-related protein 1 (LRP1) and the scavenger type B receptor CD36. LRP1 is a large endocytic receptor that is involved in lipid homeostasis, intracellular signaling, and clearance of Aβ peptides (Dieckmann et al., 2010; Adorni et al., 2019). It expressed in hepatocytes in the liver and in vascular cells, neurons, and astrocytes in the brain and PCSK9 induces its degradation in different cell types including hepatocytes and vascular cells (Ferri et al., 2012, 2016b; Canuel et al., 2013). CD36 is involved in fibrillar Aβ-mediated microglial activation and oxidized LDL uptake and elevated levels of PCSK9 stimulate CD36 expression in macrophages (Ding et al., 2018). In the brain, PCSK9 interacts with several receptors that transport cholesterol into neurons including the LDLR, the very-low-density lipoprotein receptor (VLDLR), and the apolipoprotein E receptor 2 (ApoER2) (Adorni et al., 2019).

Besides the liver, PCSK9 is expressed in the small intestine, kidney, and brain. Determining the role of PCSK9 in the brain is particularly important because while the brain is the most cholesterol-rich organ in the body, composing almost 25% of the body’s total cholesterol, its cholesterol synthesis and regulation is isolated from peripheral tissues. Neither cholesterol nor PCSK9 cross the blood-brain barrier (BBB) under normal conditions (Dietschy, 2009; Nieweg et al., 2009; Chen et al., 2014); however, several disease states can cause BBB permeability and leakage that might affect brain cholesterol homeostasis. In a human study of CNS PCSK9 concentrations, average cerebral spinal fluid (CSF) PCSK9 concentration was 5 ng/ml and remained constant over 24 h, while average serum PCSK9 concentrations were diurnal and varied from 183 ng/ml in the afternoon to 552 ng/ml in the early morning (Chen et al., 2014). Because cholesterol homeostasis is separate in the brain, it is necessary to determine the specific role of PCSK9 in the nervous system. In addition, recent data show that PCSK9 is dynamically regulated and more highly expressed in different neuropsychiatric disease states.

## PCSK9 Role in the Brain

### Neuronal Differentiation

**FIGURE 1:**
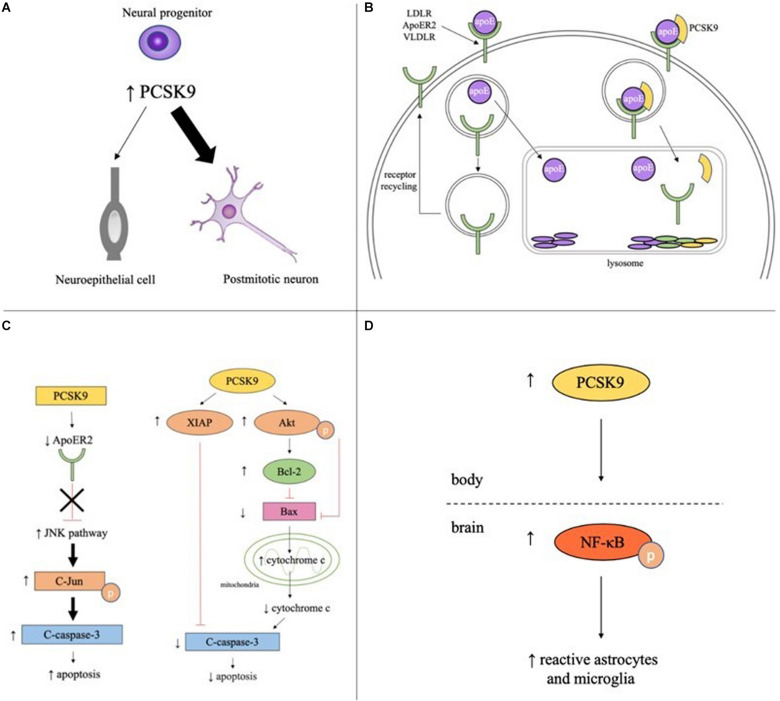
Potential roles of PCSK9 in neuronaldifferentiation, cholesterol regulation, apoptosis, and inflammation in the brain. **(A)** PCSK9 may influence neuronal differentiation as elevated PCSK9 in neural progenitors increases number of postmitotic neurons and decreases number of neuroepithelial cells. **(B)** In the absence of PCSK9, apoE binds to LDLR, ApoER2, or VLDLR on the surface of neurons, the complex is endocytosed, apoE is cleared from the extracellular fluid, and the receptor is recycled back to the plasma membrane. With PCSK9, the receptor, apoE, and PCSK9 are endocytosed and the entire complex is targeted to the lysosome for degradation. **(C)** PCSK9 promotes neuronal apoptosis through the JNK pathway by decreasing ApoER2 levels and increasing phosphorylated c-Jun and cleaved caspase-3. PCSK9 promotes neuronal survival by increasing expression of anti-apoptotic proteins XIAP, phosphorylated Akt, and Bcl-2, decreasing expression of anti-apoptotic proteins Bax and cleaved caspase-3, and decreasing cytosolic cytochrome c. **(D)** Serum PCSK9 promotes neuroinflammation by increasing levels of phosphorylated NF-κB and the number of reactive astrocytes and microglia.

PCSK9 is highly expressed in cells with proliferative ability including hepatocytes, kidney mesenchymal cells, and telencephalon neurons (Seidah et al., 2003). During development, PCSK9 is detectable at the start of neurogenesis (three-somite stage, 10.33 h post fertilization) in zebrafish and during telencephalon and cerebellum neurogenesis in mice (E12.5 and E17-P15, respectively). In adulthood, PCSK9 is only expressed in areas of continued neurogenesis like cortical, intracranial, and cerebellar granule neurons in zebrafish and the rostral extension of the olfactory peduncle (RE-OP) in mice (Seidah et al., 2003; Poirier et al., 2006; Rousselet et al., 2011). PCSK9 promotes neurogenesis by driving neuronal differentiation, as the overexpression of PCSK9 in mouse embryonic neural progenitor cells resulted in an increase in the number of postmitotic neurons and a concomitant decrease in the number of undifferentiated neuroepithelial cells (Figure 1A; Seidah et al., 2003).

The mechanistic role of PCSK9 in neuronal differentiation is likely independent from its mechanistic role in LDLR metabolism. Endogenous *PCSK9* mRNA levels increased sevenfold with neuroectodermal induction by retinoic acid (RA) in mouse P19 embryonal carcinoma cells but LDLR protein levels remained constant (Poirier et al., 2006). Furthermore, PCSK9 expression during neurogenesis is not controlled by transcription factors involved in cholesterol regulation. In the liver, *PCSK9* transcription is highly upregulated by sterol regulatory element-binding protein 2 (SREBP-2), a membrane-bound transcription factor that activates expression of genes encoding enzymes involved in cholesterol synthesis (Maxwell et al., 2003). Unlike *PCSK9*, *SREBP-2* mRNA expression is not changed by RA, suggesting the increase in PCSK9 expression in these neuroectodermal cell derivatives is regulated by a different mechanism than PCSK9 expression in other cholesterogenic organs like the liver (Poirier et al., 2006).

### LDL Receptor Family Metabolism

PCSK9 increases cholesterol levels in the developing brain by promoting lysosomal degradation of the LDL family of receptors (Figure 1B). These receptors bind apolipoprotein E (apoE), the principal cholesterol carrier in the brain, and transport apoE-bound cholesterol out of the pericellular fluid and into neurons, thus lowering cholesterol levels. In *Pcsk9^–/–^* mice, LDLR protein levels were significantly higher in the telencephalon at E12.5 and cerebellum at P7 than wild type (WT) mice and levels of untruncated apoE were ∼25% lower (Rousselet et al., 2011).

*In vitro* and *in vivo* studies in adult mice offer contradictory evidence on whether PCSK9 in the brain targets LDLR, VLDLR, and ApoER2 for degradation. Co-transfection of cultured HEK293 cells with LDLR, VLDLR, ApoER2, and PCSK9 resulted in a substantial decrease in all three receptor protein levels compared to the PCSK9 empty vector control (Poirier et al., 2008). *Pcsk9^–/–^* adult mice, however, did not have significantly different levels of LDLR and apoE proteins in the RE-OP, olfactory bulb, or CSF compared to WT mice despite colocalization of *PCSK9* and *LDLR* mRNA in the RE-OP (Rousselet et al., 2011). Additionally, PCSK9 overexpression or deletion did not affect LDLR, VLDLR, or ApoER2 levels in the hippocampus and cortex of the adult mouse brain (Liu et al., 2010).

One explanation for the discrepancy between *in vitro* and *in vivo* results in the adult brain is that the role of PCSK9 in cholesterol regulation may be cell- and/or tissue-specific. Another explanation is that given endogenous PCSK9 concentrations in adulthood are much lower than during development, changes in PCSK9 levels may not be sufficient to alter receptor levels. For example, following ischemic stroke induced by transient middle cerebral artery occlusion (tMCAO), PCSK9 expression significantly increased on the lesioned side of the dentate gyrus compared to the non-lesioned side in WT mice. Although LDLR protein levels were reduced in both WT and *Pcsk9^–/–^* mice after ischemic stroke, the decrease in LDLR levels was attenuated by 50% in *Pcsk9^–/–^* mice, suggesting that PCSK9 is necessary for LDLR degradation (Rousselet et al., 2011). These findings support the idea that PCSK9 does regulate LDLR levels in the adult brain, but changes are only detectable when PCSK9 levels are significantly increased because of a pathology like stroke.

### Apoptosis

PCSK9 was first discovered in a cellular model of apoptosis and subsequent models show PCSK9 confers both pro- and anti-apoptotic effects (Chiang et al., 2001; Bingham et al., 2006). PCSK9 promotes apoptosis in potassium-deprived cerebellar granule neurons (CGNs), with overexpression of PCSK9 inducing cell death and silencing of PCSK9 limiting cell death. Additionally, PCSK9 exhibits pro-apoptotic properties in other apoptotic models including staurosporine (STS)-induced CGNs and nerve growth factor-deprived dorsal root ganglion neurons (Kysenius et al., 2012). PCSK9 promotes survival in human neuroglioma U251 cells, as cells where PCSK9 was silenced exhibited apoptotic characteristics including cell shrinkage, membrane integrity loss, nuclear fragmentation, and chromatin compaction, while cells where PCSK9 was overexpressed had normal morphology (Piao et al., 2015).

PCSK9 has been proposed to promote cell death through the extrinsic and intrinsic apoptotic pathways and likely acts through the JNK pathway. In the potassium-deprived CGN model, CGNs with PCSK9 inhibited by RNA interference (RNAi) had lower levels of two pro-apoptotic proteins, phosphorylated c-Jun, which is required for JNK-dependent apoptosis, and cleaved caspase-3, a major executioner of apoptosis (Bingham et al., 2006; Kysenius et al., 2012). Interestingly, ApoER2 has been shown to promote neuronal survival by inactivating the JNK pathway (Hoe et al., 2005). ApoER2 levels increased 41% in PCSK9 RNAi CGNs compared to control cells. A knockdown of ApoER2 in PCSK9 RNAi CGNs increased the previously lower levels of cleaved caspase-3, suggesting PCSK9 mediates apoptosis at least in part through controlling ApoER2 levels (Figure 1C; Kysenius et al., 2012). In the STS-induced CGN model of apoptosis, PCSK9 RNAi reduced caspase-3 activation in CGNs but had no effect on phospho-c-Jun activity, suggesting PCSK9 may promote apoptosis through JNK-independent pathways as well (Kysenius et al., 2012).

PCSK9 mediates neuroglioma U251 cell survival through the intrinsic, or mitochondrial, apoptotic pathway. PCSK9 small interfering RNA (siRNA) increased activation of the pro-apoptotic protein caspase-3, downregulated anti-apoptotic proteins like XIAP and p-Akt, and increased the ratio of Bax/Bcl-2 leading to the increased release of cytochrome c from mitochondria into the cytosol. PCSK9 overexpression had the opposite effect, decreasing the amount of cleaved caspase-3, the ratio of Bax/Bcl-2 and the amount of cytochrome c release, and increasing the amount of XIAP and p-Akt present in the cells (Figure 1C; Piao et al., 2015). While the pathways by which PCSK9 regulates apoptosis have been elucidated, the exact mechanism by which PCSK9 changes concentration levels of the different proteins in the pathways is still unclear and more studies are required to determine whether PCSK9 is acting directly on these proteins or through downstream effects of the signaling pathway.

Similar to LDLR metabolism, *in vitro* results were not observed *in vivo* (Liu et al., 2010; Kysenius et al., 2012). Administration of the PCSK9 inhibitor (PCSK9i) Prep2−8 trifluoroacetate salt before, during, or after cardiac ischemia/reperfusion injury (I/R; left anterior descending coronary artery ligation) did not affect the percentage of apoptotic cells, measured by the TUNEL assay, or the levels of Bax and Bcl-2 in the rat brain (Apaijai et al., 2019). Additionally, *PCSK9* mRNA expression was not observed in the infarct or the penumbra of the hippocampus after tMCAO in mice, suggesting PCSK9 does not play a significant role in neuronal death in rodent models (Rousselet et al., 2011).

### Neuroinflammation

More recently, studies have shown that PCSK9 may promote neuroinflammation. A rat model of cardiac I/R injury revealed increased levels of p-NFκB/NFκB and activation of astrocytes and microglia. Intravenous administration of the PCSK9i Prep2−8 trifluoroacetate salt significantly reduced p-NFκB expression and rescued the reactive microglial and astrocytic proliferation and hypertrophy phenotypes induced by cardiac I/R injury (Figure 1D; Apaijai et al., 2019). Of interest, the PCSK9i did not reduce PCSK9 levels in the brain, suggesting the PCSK9i did not cross the BBB and was not acting directly on the brain. These results imply that inhibition of PCSK9 reduces brain inflammation by lowering serum PCSK9 concentrations and modulating systemic inflammation. PCSK9 may also play a part in local neuroinflammation by controlling LDLR and apoE levels. A study in BV2 microglia and human THP-1 monocytes found apoE and apoE mimetics reduced LPS-mediated TNFα and IL-6 secretion and p44/42 MAPK, JAK2, and STAT3 phosphorylation by interacting with LDLRs (Wang et al., 2019). More studies are needed to determine the extent to which PCSK9 acts systemically and locally to control inflammation and immunity in the brain.

## PCSK9 and Disease

### Neural Tube Defects

**FIGURE 2:**
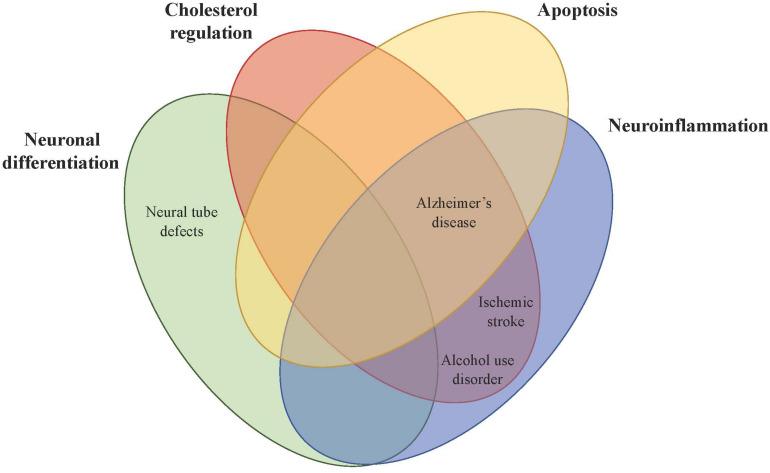
Theorized mechanistic roles of PCSK9 in central nervous system disorders. PCSK9 affects brain physiology directly or indirectly in four main areas (neuronal differentiation, cholesterol regulation, apoptosis, and neuroinflammation), which then impacts CNS disorders including neural tube defects, Alzheimer’s disease, alcohol use disorder, and ischemic stroke.

Given the importance of PCSK9 in neuronal differentiation, low maternal PCSK9 serum levels are associated with fetal neural tube defects (NTDs) (Figure 2). PCSK9 protein levels were reduced in pregnant rats with retinoic acid-induced spina bifida aperta (SBA) fetuses compared to normal pregnant rats. The same trend was observed in humans, with serum PCSK9 levels in pregnant women carrying fetuses with NTDs 0.73-fold lower compared to controls throughout gestation. Researchers have proposed using PCSK9 as a non-invasive biomarker for prenatal NTDs and diagnostically it has a sensitivity of 56.67% and a specificity of 98% (An et al., 2015).

Along with NTDs, PCSK9 is necessary for overall survival in certain species. A knockdown of PCSK9 in zebrafish eggs resulted in defective neurogenesis, absence of midbrain-hindbrain boundary, and lethality (Poirier et al., 2006). Interestingly, PCSK9 silencing is not lethal in mammals like mice or humans. Total *Pcsk9^–/–^* mice were viable and had normal organization of the telencephalon and cerebellum (Rashid et al., 2005; Seidah et al., 2008; Zaid et al., 2008; Rousselet et al., 2011). A woman with loss-of-function mutations in both maternal and paternal *PCSK9* alleles has no immunodetectable circulating PCSK9 but was healthy, fertile, and college-educated (Zhao et al., 2006). The nonsense *PCSK9* mutation C679X, found in 3.7% of African women who attended clinics in Zimbabwe, lowered LDL-cholesterol by 27% but did not adversely affect patients’ development or health (Hooper et al., 2007). These differences in survival between species suggest mammals may have compensatory mechanisms for PCSK9 silencing, such as other proprotein convertases, that fish do not have.

### Alzheimer’s Disease

Given the role of PCSK9 in apoptosis, lipoprotein receptor metabolism, and inflammation, PCSK9 might play a regulatory role in Alzheimer’s disease (AD) pathogenesis (Figure 2). Neuronal cell death causes AD and PCSK9 has been shown to exhibit a pro-apoptotic effect in several cellular models by degrading ApoER2, which confers neuronal survival (Beffert et al., 2006; Kysenius et al., 2012). Polymorphisms in the *ApoER2* gene are associated with AD risk and adult *ApoER2^–/–^* mice had an accelerated loss of corticospinal neurons during normal aging (Ma et al., 2002).

PCSK9 expression is also associated with β-site amyloid precursor protein-cleaving enzyme 1 (BACE1) expression, the enzyme that cleaves amyloid precursor protein (APP) into toxic amyloid β (Aβ). Overexpressing PCSK9 in human neuroglioma (H4) cells reduced levels of mature and immature forms of BACE1, while downregulating PCSK9 with siRNA increased expression of BACE1 and total Aβ deposition in Chinese hamster ovary (CHO) cells (Jonas et al., 2008). Conversely, inhibiting PCSK9 in a rat model of stroke resulted in attenuated Aβ aggregation, suggesting PCSK9 promotes plaque formation (Apaijai et al., 2019). One way PCSK9 promotes amyloid plaque formation may be by targeting LRP1 and CD36, the two main lipoprotein receptors involved in Aβ clearance (Adorni et al., 2019). Deletion of LRP1 in the brain endothelium of mice resulted in elevated soluble brain Aβ, reduced plasma Aβ levels, and spatial learning and memory deficits, suggesting LRP1 is important in the systemic elimination of Aβ via the BBB (Storck et al., 2016). CD36 is expressed in microglia and enhances clearance of phagocytosis of Aβ as well as oxidized-LDL uptake (Hickman et al., 2008). Other authors report PCSK9 does not regulate BACE1 or Aβ levels in mice and more work is needed to elucidate the molecular role of PCSK9 in AD (Liu et al., 2010; Fu et al., 2017).

More generally, elevated plasma LDL-C levels are associated with higher probability of early-onset AD (Zambón et al., 2010; Wingo et al., 2019) and vascular dementias (Chung et al., 2019). While there is almost no exchange of LDL particles between the CNS and periphery because of the BBB, high serum LDL may increase Aβ plaque formation in the brain by changing the balance of oxysterols or by weakening the BBB through inflammatory mechanisms (Reed et al., 2014). Side-chain oxidized cholesterol metabolites 24S-hydroxycholesterol (24-OH), which is synthesized in the brain and important for cholesterol elimination, and 27-hydroxycholesterol (27-OH), which is mostly synthesized in peripheral tissues, are able to pass the BBB. 27-OH levels increase with LDL-C levels and a change in the balance of 24-OH and 27-OH in the brain due to increased plasma LDL-C from PCSK9 may promote amyloidosis (Björkhem et al., 2009). Additionally, serum hypercholesterolemia may promote inflammation that damages the BBB and allows passage of LDL, pro-inflammatory cytokines, and other factors into the brain that increase Aβ aggregation (Altman and Rutledge, 2010).

It is also possible that PCSK9 promotes neuroinflammation and AD by mediating glucose tolerance and type 2 diabetes mellitus risk. *Pcsk9^–/–^* mice had impaired insulin secretion leading to accumulation of insulin in beta cells and irregular islet morphology due to increased LDLR expression in the pancreas (Da Dalt et al., 2018). Impaired insulin signaling promotes AD pathogenesis in the brain through increased tau phosphorylation, pro-inflammatory cytokine production, and oxidative stress (Akhtar and Sah, 2020).

Brain autopsies reveal elevated *PCSK9* mRNA and protein levels in the frontal cortices of late-onset AD patients compared to controls (Picard et al., 2019). CSF PCSK9 levels were increased and positively correlated with apolipoprotein levels in AD patients (apoE4) and cognitively normal subjects at risk for AD (apolipoprotein B, apoE, and apolipoprotein J), indicating PCSK9 dysregulation may be evident before onset of AD. Increased CSF PCSK9 is also correlated with specific AD biomarkers including amyloid β (Aβ_42_), phospho Tau (P-tau), and total Tau (T-tau) (Zimetti et al., 2017; Picard et al., 2019). Another study found that CSF PCSK9 levels did not differ between AD and non-AD controls, but that PCSK9 levels were increased in patients with neurodegenerative disorders more broadly. AD patients and non-AD controls with neurogenerative disorders exhibited significantly higher PCSK9 compared to patients and controls without neurodegenerative disorders (Courtemanche et al., 2018). Additionally, CSF PCSK9 was positively correlated with AD biomarkers including Aβ_1__–__42_, P-tau, and T-tau independent of AD (Cariou et al., 2017; Courtemanche et al., 2018).

Loss-of-function (LOF) *PCSK9* polymorphisms that cause significantly lower cholesterol concentrations are not associated with AD incidence and may even be protective against AD (Supplementary Table 1). The LOF small nucleotide polymorphism (SNP) rs11583680 was not correlated with the onset of AD in Japanese patients (Shibata et al., 2005). The most common *PCSK9* LOF mutations in French Canadian individuals, InsLEU and R46L, did not have a protective or deleterious effect on AD prevalence or age of onset in French Canadian subjects (Reynolds et al., 2010; Paquette et al., 2018). Additionally, a Mendelian randomization study of 111,194 Danish individuals showed that lower LDL cholesterol levels due to *PCSK9* LOF variants rs11591147, rs148195424, and rs562556 did not increase the risk of Alzheimer’s disease, and instead may have a causal effect in reducing the risk of AD (Benn et al., 2017). Genetic studies show some gain-of-function (GOF) *PCSK9* variants are associated with AD risk in a gender-dependent manner. The GOF SNP rs505151 (E670G) has no association with AD or dementia in 111,194 Danish individuals and the SNP rs662145 was not associated with onset of AD in Japanese patients (Shibata et al., 2005; Benn et al., 2017). However, postmortem brain tissue from French Canadian individuals showed females, but not males, with the rs499718 and rs4927193 variants had significant association with late-onset AD risk (Picard et al., 2019). These studies are not entirely conclusive and future studies may need to look at compound heterozygotes to capture variability.

### Alcohol Use Disorder

Alcohol upregulates PCSK9 expression in the brain, as PCSK9 levels in the CSF of patients with alcohol use disorder (AUD) were significantly higher compared to controls. Plasma PCSK9 levels were positively correlated with CSF PCSK9 levels in patients with AUD, while CSF studies in healthy human volunteers found that there was no significant correlation between serum and CSF PCSK9 levels (Chen et al., 2014; Lee et al., 2019a). The *PCSK9* variant rs17111503 was not associated with alcohol drinking in a Han and Uygur population (Supplementary Table 1; Han et al., 2017). Although PCSK9 expression may not affect drinking behavior, alcohol has been shown to control PCSK9 expression by modulating methylation of the gene. Epigenome-wide association analysis in postmortem bulk brain tissue shows that chronic alcohol consumption is associated with various methylation sites in the *PCSK9* gene. 17 probes corresponding to 12 genes were associated with alcohol status and the salience, executive control, visual, and motor networks and the most significant gene-associated probe was located in the *PCSK9* promoter (Lohoff et al., 2018).

Increased PCSK9 expression with alcohol may also have an impact on lipid metabolism and inflammation observed with alcohol use (Figure 2). Administration of the monoclonal antibody alirocumab against PCSK9 in a rat model of chronic alcohol exposure increased LDLR protein levels and attenuated alcohol-induced inflammation in the liver. mRNA expression of pro-inflammatory cytokines and neutrophil infiltration was significantly lower in the treatment group compared to the alcohol group, and most cytokines were reduced back down to WT levels (Lee et al., 2019b). PCSK9 plays a role in systemic cholesterol regulation and inflammation in response to alcohol that may indirectly impact the brain, although more studies are needed to examine the specific molecular role of PCSK9 and alcohol in brain pathology.

### Stroke

Rodent studies show upregulation of *PCSK9* mRNA levels following transient middle cerebral artery occlusion (tMCAO) to model ischemic stroke. *PCSK9* mRNA expression was increased in the dentate gyrus but not the infarct or penumbra, suggesting PCSK9 does not play a role in neuronal apoptosis after ischemic stroke. While adult neurogenesis occurs in the dentate gyrus, PCSK9 does not appear to play a role in neuronal differentiation after ischemic stroke, as the BrdU cell proliferation assay did not reveal significant *de novo* neurogenesis in the dentate gyrus. LDLR protein levels in the hippocampus were reduced in both WT and *Pcsk9^–/–^* mice following tMCAO but the decrease was 50% less in *Pcsk9^–/–^* mice, suggesting PCSK9 promotes LDLR metabolism after ischemic stroke (Rousselet et al., 2011). A study of brain damage in rats induced by cardiac ischemic/reperfusion injury showed PCSK9 inhibition significantly reduced the number of reactive astrocytes and microglia after injury, showing PCSK9 is involved in neuroinflammation as well (Figure 2; Apaijai et al., 2019).

Genetic studies in humans report an association between ischemic stroke risk and several GOF mutations in the *PCSK9* gene that cause increased plasma LDL-C (Supplementary Table 1). The rs2479408 and rs1711503 GOF variants are significantly associated with cerebral ischemic stroke in 408 Han Chinese cerebral ischemic stroke patients and 348 controls. The rs505151 (E670G) GOF mutation was not associated with ischemic stroke risk in the same population (Han et al., 2014); however, two other studies of the same gene variant did find an association between the mutation and stroke incidence (Slimani et al., 2014; Au et al., 2015). The Belgium Stroke Study (BSS) looked at the rs505151 (E670G) GOF mutation in 237 central Europeans with small-vessel occlusion or large-vessel atherosclerosis (LVA) and found significant association of the gene variant with increased plasma LDL-C levels, severity of coronary atherosclerosis, and risk of LVA stroke (Abboud et al., 2007).

Most LOF variants in the *PCSK9* gene have no association with ischemic stroke risk (Supplementary Table 1). The Atherosclerosis Risk in Communities (ARIC) Study of atherosclerosis followed 3,363 black subjects (2.6% had mutations in *PCSK9*) and 9,524 white subjects (3.2% had mutations in *PCSK9*) over a 15-year interval and showed no difference in stroke rates between participants with *PCSK9* LOF Y142X or C679X variants and controls (Cohen et al., 2006). A meta-analysis of eight observational cohorts and one randomized trial of statin therapy found that in patients with the same LOF variants there was no association between *PCSK9* mutations and stroke incidence (Kent et al., 2017). The rs11583680 LOF variant was not associated with risk of ischemic stroke or its subtypes in 161 Han Chinese ischemic stroke patients and 483 matched controls (Zhao et al., 2019). Several studies and a meta-analysis of the loss-of-function rs11591147 (R46L) *PCSK9* variant found no association with ischemic stroke and ischemic stroke subtypes (Cohen et al., 2006; Kostrzewa et al., 2008; Hopewell et al., 2017; Kent et al., 2017). Interestingly, a Mendelian randomization study of the *PCSK9* LOF variant rs11591147 (R46L, G/T) in 337,536 individuals from the UK Biobank found the T allele was protective against ischemic stroke in the hypothesis-driven set and a nominally significant association with stroke in the full data set (Rao et al., 2018). While most studies show *PCSK9* LOF variants are not associated with ischemic stroke incidence, findings vary due to different measurements and classifications of stroke, variabilities among datasets and populations, and differences in statistical power and analyses between studies. Additionally, lower LDL-C levels are not associated with ischemic stroke risk (Pikula et al., 2015).

While low PCSK9 and LDL-C levels may not reduce baseline stroke risk, PCSK9 inhibitors (PCSK9i) help reduce stroke incidence in patients with high cholesterol and high risk of cardiovascular disease. A meta-analysis concluded that all cholesterol-lowering therapies should reduce the risk of stroke because lowering circulating cholesterol levels decreases the risk of atherosclerosis and embolic thrombus (De Caterina et al., 2016; Castilla-Guerra and Fernandez-Moreno, 2017). A study of evolocumab, a monoclonal antibody against PCSK9, showed that stroke is significantly reduced in the group that received the drug compared to the group that received placebo in a similar magnitude to statins when treated over a period of 2 years (Sabatine et al., 2017). Other analyses of PCSK9 inhibitors, however, found that there were no associations with stroke reduction. The Open-Label Study of Long-term Evaluation Against LDL-C (OSLER) and The Long-term Safety and Tolerability of Alirocumab in High Cardiovascular Risk Patients with Hypercholesterolemia Not Adequately Controlled with Their Lipid Modifying Therapy (ODYSSEY LONG TERM) are phase 2 and 3 safety studies of evolocumab and alirocumab, respectively, that found no significant effect of these PCSK9i on stroke rate, even when transient ischemic attacks were included in the analysis. The number of patients, 4,465 in OSLER and 2,341 in ODYSSEY LONG TERM, were relatively small and the study period, 1 year and 1.5 years, respectively, were relatively short, so longer studies are needed to fully evaluate effect of evolocumab and alirocumab on stroke incidence (Koren et al., 2014; Robinson et al., 2015; Milionis et al., 2016).

Hemorrhagic stroke was rarely reported in genetic studies given its rarity. One study observed no association between PCSK9 and hemorrhagic stroke (Rao et al., 2018). A meta-analysis of 23 studies found that low LDL-C levels were associated with hemorrhagic stroke but theorized it may be due to patients’ poor health status in general rather than a causative role PCSK9 or low LDL-C (Kim et al., 2009; Wang et al., 2013; Kent et al., 2017).

## PCSK9 Inhibitors and Neurocognition

In 2012, the FDA warned of potential non-serious and reversible cognitive side effects such as memory loss, forgetfulness, and confusion related to cholesterol-lowering statin drugs (Parker et al., 2010; Gauthier and Massicotte, 2015). Despite case reports of neurocognitive impairment with statins, meta-analyses and longitudinal studies in larger populations suggest statins do not increase cognitive impairment risk and may slow the rate of AD in some individuals (Beydoun et al., 2011; Bettermann et al., 2012; Ott et al., 2015). Given the effect of PCSK9 on cholesterol levels and the importance of cholesterol regulation in brain function, there was a question of whether PCSK9 inhibition would also have adverse neurocognitive effects.

In 2015, the FDA approved two PCSK9i, evolocumab and alirocumab, to treat hypercholesterolemia. Evolocumab, an IgG2 isotype, and alirocumab, an IgG1 isotype, are fully human monoclonal antibodies that interact with circulating PCSK9 to prevent it from binding to LDLRs, thus reducing LDLR degradation and lowering plasma LDL cholesterol by 50–60% (Chaudhary et al., 2017; Nishikido and Ray, 2018). Although monoclonal antibodies do not typically cross the intact BBB, early phase 2 safety studies reported a non-significant trend in neurocognitive impairment with PCSK9i (Swiger and Martin, 2015). The OSLER study found neurocognitive events such as amnesia or mental impairment occurred in 0.9% of those given evolocumab and 0.3% of those in the standard of care group without evolocumab (Koren et al., 2014). Reports of adverse events may have been skewed because OSLER was unblinded, no objective neurocognitive measures were performed, and those receiving evolocumab had more in-person visits and thus more opportunity to report cognitive changes (Swiger and Martin, 2015). The ODYSSEY LONG TERM trial using alirocumab showed memory impairment in 1.2% of the alirocumab group and 0.5% of the placebo group, but the difference between the groups was not statistically significant (Robinson et al., 2015).

Phase 3 clinical trials with larger sample sizes and longer follow-up periods found that there are not significant neurocognitive adverse events associated with PCSK9 inhibitors. The Evaluating PCSK9 Binding antiBody Influence oN coGnitive HeAlth in High cardiovascUlar Risk Subjects (EBBINGHAUS) is a subset of the FOURIER phase 3 clinical trial of evolocumab (Giugliano et al., 2017a; Sabatine et al., 2017). EBBINGHAUS followed 1,204 patients over 26 months and used the Cambridge Neuropsychological Test Automated Battery (CANTAB) to measure neurocognition. There was memory or concentration difficulty in 1.9% of the evolocumab group and 1.6% of the placebo group. This difference was not significant and there was no association between PCSK9i or low LDL-C and neurocognitive decline (Giugliano et al., 2017b). Patients in the EBBINGHAUS trial were still followed for a relatively short period of time, which may limit definite conclusions about the long-term neurocognitive effects of PCSK9i. Another clinical trial to evaluate neurocognitive function using CANTAB with long-term exposure to alirocumab is estimated to be completed in March 2020 (NCT02957682) (Mannarino et al., 2018)^1^. A recent meta-analysis of 14 randomized trials also found no change in neurocognition with PCSK9i (Robinson et al., 2017).

Furthermore, there were no neurocognitive changes observed in people with *PCSK9* polymorphisms. REasons for Geographic and Racial Differences in Stroke (REGARDS) was a prospective cohort study of the association between *PCSK9* loss-of-function variants (C697X or Y142X) and neurocognitive impairment and decline in 10,695 black individuals over 5.6 years. Verbal learning, verbal memory, semantic fluency, and global cognitive function of 241 participants with and 10,454 without LOF variants were evaluated by tests from the Consortium to Establish a Registry for Alzheimer’s Disease (CERAD) battery and the Six-Item Screener (SIS) assessment. The REGARDS study found that there was no association between *PCSK9* LOF variants and neurocognitive impairment or decline over time in blacks. Furthermore, while participants with *PCSK9* LOF variants had significantly lower LDL-C compared to those without polymorphisms, there was no difference in CERAD and SIS scores between the two groups (Mefford et al., 2018). Several studies of other LOF *PCSK9* variants including rs11591147 (R46L) and rs639750 in diverse groups such as elderly, British, and African-ancestry individuals also found no association with neurocognitive disorders (Postmus et al., 2013; Rao et al., 2018; Verbeek et al., 2018; Safarova et al., 2019). These results show that lifelong exposure to low PCSK9 levels and corresponding low LDL-C levels do not have a major effect on longitudinal changes in neurocognition and are consistent with neurocognitive outcomes from the EBBINGHAUS study.

Mendelian randomization (MR) studies looked at other potential neuropsychiatric effects associated with *PCSK9* silencing. PCSK9 was nominally associated with depression in an MR analysis of the LOF *PCSK9* gene variant rs1159147 T allele in 479,522 UK Biobank individuals (Nelson et al., 2019). A second MR study conducted based on summary statistics from genome-wide association studies found a statistically significant increased risk of depression after correcting for multiple testing with PCSK9i treatment. PCSK9 was not found to impact insomnia or neuroticism (Alghamdi et al., 2018).

## Conclusion

Despite the well-researched role of PCSK9 in the liver, the role of PCSK9 in the brain is still unclear, though rapidly emerging. *In vitro* and *in vivo* studies suggest PCSK9 is involved in the differentiation of neural progenitor cells to neurons, the targeting of receptors in the LDLR family to lysosomal degradation, the regulation of neuronal apoptosis, and the activation of astrocytes and microglia in the brain. Cell lines, animal models, and genetic studies reveal the role of PCSK9 in several CNS diseases including Alzheimer’s disease, alcohol use disorder, ischemic stroke, and neuropsychiatric disorders. One important question that remains to be answered is if in these diseases PCSK9 has a local effect on the brain or a systemic effect in peripheral tissues that then affects the brain. For example, in Alzheimer’s disease PCSK9 plays a direct role in the brain by lowering BACE1 expression and an indirect role by increasing LDL-C levels, which affects Aβ plaque formation and aggregation. Similarly, it is unclear whether PCSK9 has a systemic effect on inflammation by elevating plasma LDL-C levels, or if PCSK9 also acts locally in the brain to control inflammation. Future work is also needed to explore the systemic vs. localized brain effects of PCSK9 monoclonal antibodies and if or to what extent these antibodies cross the BBB during disease states including AD, stroke, and chronic inflammatory brain diseases.

One potential method to explore the effect of PCSK9 in the brain is by using inclisiran, a novel PCSK9 inhibitor. Inclisiran is a long-acting, synthetic small interfering RNA (siRNA) that blocks the synthesis of PCSK9 by degrading *PCSK9* mRNA using the body’s natural pathway of RNA interference. Inclisiran associates with the RNA-induced silencing complex (RISC) inside the cell and directs RISC to cleave *PCSK9* mRNA catalytically, with one enzyme cleaving several transcripts, lowering the number of transcripts available for protein translation and decreasing the concentration of PCSK9 protein. Inclisiran uptake is specifically targeted to hepatocytes by conjugating the siRNA to triantennary N-acetylgalactosamine carbohydrates that bind to liver-expressed asialoglycoprotein receptors (Fitzgerald et al., 2016; Kosmas et al., 2018). Clinically, phase 2 and 3 trials show inclisiran reduces LDL-C levels by ~50% with relatively benign side effects and only requires one subcutaneous injection every 6 months compared to injections every 2 weeks for monoclonal antibody PCSK9i (Ray et al., 2017, 2020). Unlike monoclonal antibody PCSK9i, inclisiran also has the potential to specifically inhibit PCSK9 in the brain. While naked siRNA cannot cross the BBB, siRNA can be targeted to the brain through receptor-mediated transcytosis (Zheng et al., 2018). The rabies virus glycoprotein (RVG29) ligand has been used to target siRNA nanomedicines to the brain in several diseases including AD, Parkinson’s disease and traumatic brain injuries (Kumar et al., 2007; Cooper et al., 2014; Park et al., 2015; Kwon et al., 2016; Zheng et al., 2018). In the future, inclisiran may be targeted to the brain and used as both a novel research tool and a promising therapeutic.

## Author Contributions

EO’C and FL wrote the manuscript. EO’C designed the figures and table with the mentorship, guidance, and editing of FL.

## Conflict of Interest

The authors declare that the research was conducted in the absence of any commercial or financial relationships that could be construed as a potential conflict of interest.

## Footnotes

1 https://clinicaltrials.gov/ct2/show/NCT02957682
