# Supplementary material for: Proprotein Convertase Subtilisin/Kexin Type 9 (PCSK9) in the Brain and Relevance for Neuropsychiatric Disorders
Source: Front Neurosci. 2020 Jun 12;14:609. doi: 10.3389/fnins.2020.00609 (PMC7303295; doi:10.3389/fnins.2020.00609)
Supplement: Supplementary file 1 [file Table_1.DOCX]

Supplementary Material

**Table 1. CNS disease and *PCSK9* gene variant associations.**

| CNS Disease | PCSK9 variant | Effect | Sample | Association | Author |
| --- | --- | --- | --- | --- | --- |
| Alzheimer’s disease (AD) | rs11583680  C>G/C>T | LOF | 218 Japanese AD patients, 114 age and sex-matched controls | No association with onset of AD | (Shibata et al., 2005) |
|  | rs11591147 (R46L)  G>T | LOF | 111,194 Danish individuals from two prospective general population studies: the Copenhagen General Population Study and the Copenhagen City Heart Study | No association with AD, vascular dementia or any dementia | (Benn, Nordestgaard, Frikke-Schmidt, & Tybjærg-Hansen, 2017) |
|  |  |  | 878 participants from Quebec Founder Population (QFP) | No association with AD prevalence or age of onset | (Paquette et al., 2018) |
|  | rs148195424 (R237W)  C>T | LOF | 111,194 Danish individuals from two prospective general population studies: the Copenhagen General Population Study and the Copenhagen City Heart Study | No association with AD, vascular dementia or any dementia | (Benn et al., 2017) |
|  | rs562556 (I474V)  G>A | LOF | 111,194 Danish individuals from two prospective general population studies: the Copenhagen General Population Study and the Copenhagen City Heart Study | No association with AD, vascular dementia or any dementia | (Benn et al., 2017) |
|  | InsLEU (c.43_44insCTG or c.61_63dupCTG (L10) and c.61_63triCTG (L11)) | LOF | 878 participants from QFP | No association with AD prevalence or age of onset | (Paquette et al., 2018) |
|  | rs505151 (E670G)  G>A | GOF | 111,194 Danish individuals from two prospective general population studies: the Copenhagen General Population Study and the Copenhagen City Heart Study | No association with AD, vascular dementia or any dementia | (Benn et al., 2017) |
|  | rs662145  C>T | GOF | 218 Japanese AD patients, 114 age and sex-matched controls | No association with onset of AD | (Shibata et al., 2005) |
|  | rs499718  T>A/T>C | Intron variant | 65 late onset AD and 45 control postmortem brain frontal cortices and cerebella from QFP | Significant association with late onset AD risk in females only (p = 0.000856) | (Picard et al., 2019) |
|  | rs4927193  T>C | Intron variant | 65 late onset AD and 45 control postmortem brain frontal cortices and cerebella from QFP | Significant association with late onset AD risk in females only (p = 0.000626) | (Picard et al., 2019) |
|  |  |  | 384 individuals in Alzheimer Disease Neuroimaging Initiative (ADNI) dataset | Weak association (p=0.0974) for CSF pTau but significant association with CSF Tau (p=0.0302) in females only | (Picard et al., 2019) |
| Alcohol use disorder | rs17111503  A>G | Upstream transcript variant | 237 Han and Uygur lacunar infarction patients and 240 healthy controls | No association with alcohol drinking | (D.-f. Han et al., 2017) |
| Ischemic stroke (IS) | rs11591147 (R46L)  G>T | LOF | 9,524 white Americans from Atherosclerosis Risk in Communities (ARIC) study | No association with risk of IS or its subtypes | (Cohen, Boerwinkle, Mosley, & Hobbs, 2006) |
|  |  |  | 176 carriers with 6,618 non-carriers | No association with risk of IS or its subtypes | (Kostrzewa, Broda, Kurjata, Piotrowski, & Ploski, 2008) |
|  |  |  | 10,307 European IS cases and 19,326 controls | No association with risk of IS or its subtypes | (Hopewell et al., 2017) |
|  |  |  | 31,306 whites with 955 (3.1%) having at least one R46L variant | No association with risk of IS or its subtypes | (Kent et al., 2017) |
|  |  |  | 337,536 British individuals from UK Biobank | T allele protective against IS (OR = 0.61±0.18, p = 2.40×10^−3^) | (Rao Abhiram et al., 2018) |
|  | rs11583680  C>G/C>T | LOF | 161 Han Chinese patients with IS, 483 matched controls | No association with risk of IS or its subtypes | (Zhao, Li, Lei, Huang, & Yang, 2019) |
|  | rs67608943 (Y142X)  C>G/C>T    or  rs28362286 (C679X)  C>A/C>T | LOF | 3,363 African Americans from Atherosclerosis Risk in Communities (ARIC) study | No association with risk of IS or its subtypes | (Cohen et al., 2006) |
|  |  |  | 17,459 African Americans (AAs) with 403 (2.3%) having at least one Y142X or C679X variant | No association with risk of IS or its subtypes | (Kent et al., 2017) |
|  | rs505151 (E670G)  G>A | GOF | 408 Han Chinese cerebral ischemic stroke patients and 348 control subjects | No association with risk of IS or its subtypes | (D. Han et al., 2014) |
|  |  |  | 114 Tunisian patients with IS, 232 controls | Increased risk of IS (*p* = 0.032), G allele higher in IS subgroup | (Slimani et al., 2014) |
|  |  |  | Seven case-control studies encompassing 1,897 cases and 2,119 controls | Increased risk of IS by 36% (OR=1.36, 95% CI, 1.01-1.58) | (Au et al., 2015) |
|  | rs2479408  C>G | GOF | 408 Han Chinese cerebral IS patients and 348 control subjects | Significant association with cerebral IS (p=0.013) | (D. Han et al., 2014) |
|  | rs1711503  A>G |  | 408 Han Chinese cerebral IS patients and 348 control subjects | Significant association with cerebral IS (p=0.028) | (D. Han et al., 2014) |
| Small-vessel occlusion (SVO) or large-vessel atherosclerosis (LVA) | rs505151 (E670G)  G>A | GOF | 237 Belgian patients with SVO and LVA and 326 controls from Belgium Stroke Study | Minor allele (G) carriers associated with risk of LVA stroke (OR = 3.52, 95% CI 1.25–9.85; p = 0.017) | (Abboud et al., 2007) |
| Cognition | rs1159147  C>G/C>T | LOF | 479,522 British individuals from UK Biobank | T allele nominally associated with depression (OR, 1.089; 95% CI, 1.026–1.157; *P*=0.005) | (Nelson et al., 2019) |
|  | rs11591147 (R46L)  G>T | LOF | 5,777 elderly participants of PROspective Study of Pravastatin in the Elderly at Risk (PROSPER) | No association with cognitive performance or activities of daily living | (Postmus et al., 2013) |
|  |  |  | 337,536 British individuals from UK Biobank | No association with cognitive dysfunction | (Rao Abhiram et al., 2018) |
|  |  |  | 4,615 participants | No association with cognition | (Verbeek et al., 2018) |
|  |  |  | 1,232 EHR-derived binary phecodes in 51,700 European-ancestry (EA) individuals and 585 phecodes in 10,276 African-ancestry (AA) individuals | No association with neurocognitive disorders | (Safarova et al., 2019) |
|  | rs67608943 (Y142X)  C>G/C>T    or  rs28362286 (C679X)  C>A/C>T | LOF | 10,695 African Americans from REasons for Geographic and Racial Differences in Stroke (REGARDS) study | No association with neurocognitive effects | (Mefford Matthew et al., 2018) |
|  | rs639750  G>T | Intron variant | 1,232 EHR-derived binary phecodes in 51,700 European-ancestry (EA) individuals and 585 phecodes in 10,276 African-ancestry (AA) individuals | No association with neurocognitive disorders | (Safarova et al., 2019) |
